# Supplementary material for: Quantifying noxious-evoked baseline sensitivity in neonates to optimise analgesic trials
Source: eLife. 2021 Apr 13;10:e65266. doi: 10.7554/eLife.65266 (PMC8087440; doi:10.7554/eLife.65266)
Supplement: Transparent reporting form [file elife-65266-transrepform.pdf]

eLIFE
1st Floor
24 Hills Road
Cambridge CB2 1JP, UK
P 01223 855340
W elifesciences.org
T @elife

eLife's transparent reporting form

We encourage authors to provide detailed information *within their submission* to facilitate the interpretation and replication of experiments. Authors can upload supporting documentation to indicate the use of appropriate reporting guidelines for health-related research (see EQUATOR Network), life science research (see the BioSharing Information Resource), or the ARRIVE guidelines for reporting work involving animal research. Where applicable, authors should refer to any relevant reporting standards documents in this form. If you have any questions, please consult our Journal Policies and/or contact us: editorial@elifesciences.org.

Sample-size estimation

- You should state whether an appropriate sample size was computed when the study was being designed
- You should state the statistical method of sample size computation and any required assumptions
- If no explicit power analysis was used, you should describe how you decided what sample (replicate) size (number) to use

Please outline where this information can be found within the submission (e.g., sections or figure legends), or explain why this information doesn't apply to your submission:

Study 1 was a feasibility study to test the principles behind the paradigm presented in the paper and a power calculation was not necessary. Study 3 sample size was computed based on power calculations from simulated data in Study 2. The assumptions and parameters used in Study 2 can be found in the results and methods sections of the manuscript. Study 4 was an opportunistic study conducted before and after a change in clinical guidelines. A similar number of infants were included in the Control group (data collected prior to the guideline change) and Intervention group (data collected following the guideline change). This information can be found in the results and methods section of the submitted manuscript.

Replicates

- You should report how often each experiment was performed
- You should include a definition of biological versus technical replication
- The data obtained should be provided and sufficient information should be provided to indicate the number of independent biological and/or technical replicates
- If you encountered any outliers, you should describe how these were handled
- Criteria for exclusion/inclusion of data should be clearly stated
- High-throughput sequence data should be uploaded before submission, with a private link for reviewers provided (these are available from both GEO and ArrayExpress)

Please outline where this information can be found within the submission (e.g., sections or figure legends), or explain why this information doesn't apply to your submission:

eLife Sciences Publications, Ltd is a limited liability non-profit non-stock corporation incorporated in the State of Delaware, USA, with company number 5030732, and is registered in the UK with company number FC030576 and branch number BR015634 at the address 1st Floor, 24 Hills Road, Cambridge CB2 1JP | August 2014

Information regarding how often each experiment was performed is not included since eligible infants were studied upon voluntary agreement and informed consent obtained from parents.

The number of independent infants from which data was recorded (independent biological replicates) and used for the different studies can be found in the results section and figures 2-5 legends.

The relationship between individual responses to experimental noxious stimuli and a subsequent clinically-required heel lance presented in Study 1 was replicated in Study 3.

The research studies' inclusion and exclusion criteria are presented in the methods section – *Study design and participants*.

High-throughput sequence data was not used in the submitted manuscript.

Statistical reporting

- Statistical analysis methods should be described and justified
- Raw data should be presented in figures whenever informative to do so (typically when N per group is less than 10)
- For each experiment, you should identify the statistical tests used, exact values of N, definitions of center, methods of multiple test correction, and dispersion and precision measures (e.g., mean, median, SD, SEM, confidence intervals; and, for the major substantive results, a measure of effect size (e.g., Pearson's r, Cohen's d)
- Report exact p-values wherever possible alongside the summary statistics and 95% confidence intervals. These should be reported for all key questions and not only when the p-value is less than 0.05.

Please outline where this information can be found within the submission (e.g., sections or figure legends), or explain why this information doesn't apply to your submission:

Statistical analysis methods including the statistical tests used for all the studies are described and justified in the methods section – *Statistical analysis.* Figures 2-5 present scatter plots with raw data and individual infant's EEG responses are shown in supplementary materials. Exact values of N, t statistics and p values are reported in the results section and in the legends of figures 2-5.

(For large datasets, or papers with a very large number of statistical tests, you may upload a single table file with tests, Ns, etc., with reference to sections in the manuscript.)

Group allocation

- Indicate how samples were allocated into experimental groups (in the case of clinical studies, please specify allocation to treatment method); if randomization was used, please also state if restricted randomization was applied
- Indicate if masking was used during group allocation, data collection and/or data analysis

Please outline where this information can be found within the submission (e.g., sections or figure legends), or explain why this information doesn't apply to your submission:

Allocation of participants into experimental groups was not random. In Study 3, infants were recruited in parallel to the control or intervention group following the same inclusion and exclusion criteria and study design. Study 4 participants were included opportunistically before and after an update to our local clinical guidelines was implemented, whereby paracetamol was administered prior to rather than post-immunisations. The participants recruited before and after the guideline change constituted the control and intervention group respectively. The data acquisition was conducted by the same team of researchers for both studies.

Investigators were not blinded to group allocation during data collection and analysis. The data presented here were collected to develop and test the nociceptive sensitivity paradigm rather than investigating the efficacy of an intervention. The results presented in the last study investigating the effect of paracetamol for immunisation-evoked brain activity are preliminary and could inform the development of a protocol for a randomised clinical trial.

This information can be found in the methods, results and discussion sections.

Additional data files ("source data")

- We encourage you to upload relevant additional data files, such as numerical data that are represented as a graph in a figure, or as a summary table
- Where provided, these should be in the most useful format, and they can be uploaded as "Source data" files linked to a main figure or table
- Include model definition files including the full list of parameters used
- Include code used for data analysis (e.g., R, MatLab)
- Avoid stating that data files are "available upon request"

Please indicate the figures or tables for which source data files have been provided:

Source data to produce figures 2-5 are provided with the paper.
